# Introduction of SWCNTs as a Method of Improvement of Electrical and Mechanical Properties of CFRPs Based on Thermoplastic Acrylic Resin

**DOI:** 10.3390/polym15030506

**Published:** 2023-01-18

**Authors:** Szymon Demski, Kamil Dydek, Kinga Bartnicka, Kamil Majchrowicz, Rafał Kozera, Anna Boczkowska

**Affiliations:** Faculty of Materials Science and Engineering, Warsaw University of Technology, 141 Wołoska, 02-507 Warsaw, Poland

**Keywords:** Elium^®^, carbon nanotubes, electrical properties, mechanical properties

## Abstract

The aim of this research was to improve the electrical and mechanical properties of carbon-fibre-reinforced polymers (CFRP) based on thermoplastic acrylic resin ELIUM^®^, by introducing single-walled carbon nanotubes (SWCNTs) into their structure. The laminates were fabricated using the infusion technique of infiltrating the carbon fabric with the mixture of acrylic resin and SWCNTs. The addition of SWCNTs improved the electrical conductivity through the thickness of the laminate by several times compared to the laminate without modification. No defects or voids were observed in the structure of the fabricated nanocomposites. The introduction of SWCNTs into the CFRP structure increased the Young’s modulus, interlaminar shear strength and impact resistance. DMA analysis showed almost no change in the glass transition temperature of the fabricated SWCNT/CFRP nanocomposites compared to the reference laminate.

## 1. Introduction

In recent years, carbon-fibre-reinforced polymers (CFRPs) have been increasingly used and are replacing components made of metallic alloys. Due to their high specific strength, high specific stress, lightweight nature [1] and ease of processing, CFRPs are widely used in the automotive [2], aerospace [3] and marine [4] fields, and as components in wind turbines [5]. To achieve the required properties of CFRPs, thermoset resins are used as a matrix for structural components and epoxy resin is the one that is used most frequently. Despite their good mechanical properties, chemical erosion resistance and ease of processing [6], CFRPs with thermoset matrices are brittle and susceptible to impact damage [7,8]. Composites with a thermoset matrix are also difficult to recycle effectively [9,10] and possess low through-thickness electrical conductivity [6,11] in spite of the fact that carbon fibres are highly conductive (60,000 S/m) [6]. The disadvantages in fibre-reinforced polymers (FRPs) are caused by the polymer matrix, which possesses much lower mechanical properties than carbon fibres and acts as an insulator in resin-rich areas between the layers of fabrics [12].

Many attempts have been made to increase the impact resistance and electrical conductivity in order to improve the properties and application possibilities of CFRPs with thermoset matrices. There is an approach that includes using carbon-based nanoparticles with excellent mechanical, thermal and electrical properties [13]. Recently carbon-based nanoparticles such as fullerenes, graphene, graphene oxide, graphene nanoplates and carbon nanotubes (CNTs) have attracted researchers’ attention. CNTs possess exceptional mechanical properties (elastic moduli of 1 TPa and tensile strength of 50–200 GPa [14,15]), thermal conductivity (about twice that of diamonds [16]) and electrical conductivity (the electrical current carrying capacity is 1000 times higher than the of copper wires [16]). Two types of CNTs can be distinguished: single-walled CNTs (SWCNTs) that exhibit metallic or semiconductor electrical conductivity depending on the hexagonal lattice orientation of the nanotube central axis, and multi-walled CNTs (MWCNTs) that exhibit metallic electrical conductivity [11]. The electrical conductivity of SWCNTs can range from 10^2^ S/cm to 10^6^ S/cm, and for MWCNTs, the range varies from 10^3^ S/cm to 10^5^ S/cm [17]. SWCNTs offer higher mechanical properties, intrinsic conductivity and aspect ratio than MWCNTs [18]. Due to their high aspect ratio, the percolation threshold of SWCNTs is lower. This results in a lower viscosity increase during resin modification. To improve the electrical conductivity or mechanical properties of CFRPs with CNTs, various methods can be used. These methods can be grouped into three main categories: resin modification, fibre surface modifications (e.g., grafting, growth) and interleaving (introducing conductive material between fibre layers) [6].

The resin modification involves mixing CNTs with a liquid polymer matrix before the manufacturing process of CFRPs. The introduced CNTs must be uniformly dispersed to create a three-dimensional network of the filler through the composite in order to improve the electrical conductivity and mechanical properties. Such a phenomenon is called percolation [19,20]. Compared to fillers such as carbon black, CNTs have a high aspect ratio (above 500), which allows for a percolation threshold to be achieved at lower fractions (<1%) [11,20,21]. Higher fractions of the nanofiller can lead to a high viscosity increase, thus creating problems during the manufacturing process. Due to the small particle size and strong van der Waals interaction between nanotubes, CNTs tend to agglomerate. In order to obtain the uniform dispersion of conductive filler in polymer, methods such as mechanical/magnetic stirring [22], ultrasonication [19] or mixing with three roll mills [23] must be applied.

The previously mentioned methods of counteracting the disadvantages of CFRP are considered when the thermosets are used. However, such solutions do not include the problem of the recycling of composites. A different approach can be considered when changing the polymer matrix to the thermoplastic polymer. Elium^®^, which is the novel methyl methacrylate (MMA) thermoplastic resin introduced by Arkema, seems to be an excellent proposition for such a material. This thermoplastic resin is in the liquid form at room temperature and can be processed by the methods typical for thermosets, e.g., infusion [24], pultrusion [25], compression, casting and filament winding. This resin is available in several grades with slightly different properties and dedicated methods of processing. In some cases, different grades of resin possess unique features. The Elium^®^ resin grades dedicated to the infusion process (188 XO and 188 O) have low density (1.01 g/cm^3^) and low viscosity (100 mPa·s). As a thermoplastic material, this resin undergoes free radical polymerisation with benzoyl peroxide used as an initiator. Furthermore, Elium^®^ resin is styrene- and Bisphenol A (BPA)-free and can be recycled in two ways: mechanically by grinding and compounding or chemically by the depolymerisation process.

The literature findings show that researchers have mainly focussed on the mechanical performance of Elium^®^ resin such as impact [26,27,28,29], tensile [25,30], flexure [31,32] and weldability [33,34]. On the basis of this research, it has been found that Elium^®^ resin exhibits the thermomechanical and mechanical performance comparable to the frequently used epoxy resin systems [24]. However, as a thermoplastic polymer, Elium^®^ possesses a lower glass transition temperature (T_g_) than thermoset resins and has low electrical and thermal conductivity. Due to these properties, the applications of Elium^®^ can be limited. Some research has focussed on topics such as the development of insulation materials [35], introducing phase change materials into CFRPs [36] or crack healing [37] to discover new applications for this material.

Despite a high interest in Elium^®^ resin, none of the research has focussed on improving electrical conductivity by introducing CNTs into the polymer. This article aims to present the improvement in the electrical conductivity and mechanical properties of CFRPs manufactured with the resin infusion process by introducing SWCNTs into the novel thermoplastic Elium^®^ resin. The use of thermoplastic resin that exhibits thermomechanical and mechanical performance comparable to the commonly used epoxy resin systems is a proposed solution to the problems of brittleness and recycling attributed to CFRPs with a thermoset matrix. The addition of electrically conductive filler can also improve the performance of manufactured composites and expand their applications further. Thus far, the solution mentioned above and presented in this work has not been investigated.

## 2. Materials and Methods

### 2.1. Materials

CFRPs were manufactured using ±45° stitched carbon fabric with universal sizing and an areal weight of 600 g/m^2^ (Saertex, Germany). Elium^®^ 188 O (Arkema, Colombes, France), a novel thermoplastic acrylic resin, was used as a polymer matrix. This novel resin has a low viscosity of 100 mPa·s, a density of 1.01 g/cm^3^ and can be processed by the methods commonly used for epoxy resins such as resin infusion. Dibenzoyl peroxide (Acros Organics, Geel, Belgium) was used to initiate the polymerisation process in resin. Tuball™ SWCNTs (OCSiAl, Leudelange, Luxembourg) were used as a conductive nanofiller to produce nanocomposites. The average diameter of SWCNTs was <2 nm, the length >1 μm and its purity was 75%.

### 2.2. Fabrication Methods

The Elium^®^/SWCNT mixtures were produced by mixing resin with the following SWCNTs concentrations: 0.0075 wt.%, 0.01 wt.%, 0.015 wt.% and 0.02 wt.%. After mechanical mixing, Elium^®^/SWCNT mixtures were treated with ultrasonic waves, which is a commonly used method for CNTs’ dispersion [19,38]. The ultrasonication was performed using the VCX1500 (Sonics & Materials, Newtown, CT, USA) ultrasonic processor for 1 h at the frequency of 20 kH and the amplitude of 40%. Ultrasonic waves were on for 9 s out of every 14 s of the cycle to prevent the mixture temperature from increasing over 45 °C. After ultrasonication, nanocomposites were mixed with 2 wt.% of initiator and put in a mould to prepare samples.

CFRPs with a ply stacking sequence of [±45°]_4s_ and dimensions of 300 mm × 300 mm were manufactured by resin infusion. The neat Elium^®^ resin and mixtures with different concentrations of SWCNTs were used as matrix materials. The manufacturing process was conducted at the ambient temperature and under a vacuum pressure of −0.9 bar during the whole process. Laminates were demoulded after 24 h and then post-cured at 80 °C for 2 h.

### 2.3. Measurements Methods

The viscosity measurements of neat Elium^®^ resin and Elium^®^/SWCNT mixtures were conducted using DV-II+Pro Viscometer (Brookfield, Toronto, ON, Canada). The samples of 8 mL volume were prepared after the ultrasonication process and measurements were conducted at 25 °C from 1 to 200 rpm using the SC4-21 spindle type. The spindle had a fixed shear rate value of 0.93 N per 1 rpm.

The electrical conductivity of nanocomposites and fabricated CFRPs was measured through the sample thickness (Z direction). The test samples with the dimensions of 10 mm × 10 mm were taken from different sections of casted samples and CFRPs. Five test samples represented each material. The electrical conductivity was measured using the Keithley 6221/2182A device equipped with a measuring stand with copper electrodes. The silver paste (CW7100 from Chemtronics^®^, Kennesaw, GA, USA) was used in order to ensure better contact between electrodes and a sample.

The microstructure observations of manufactured composites were performed using a Scanning Electron Microscope (SEM, SU-70 Hitachi, Tokyo, Japan). The samples of 10 × 10 mm dimensions were sanded using papers of 7 types of graininess: P80, P240, P600, P800, P1200, P2000 and P4000 and polished using 2 diamond slurries with 1 µm and 0.25 µm particle sizes. The samples were then coated with a 3 nm Au–Pd electroconductive layer using 2 kV voltage, 15 mA current and 80 s time. The observations of the prepared samples were made at an acceleration voltage of 5 kV.

The interlaminar shear strength was determined through a three-point bending of short-beam test (ILSS) specimens with the dimensions of 2.4 × 6.4 × 25 mm (thickness × width × length). Five test specimens represented each test. The short-beam tests were conducted on a static testing machine MTS QTest equipped with a 10 kN load cell and the test speed was 1 mm/min. A loading nose with the diameter of 6 mm and side supports with a diameter of 3 mm were used while the span length was set at 9.6 mm. The short-beam strength (F^sbs^) was calculated according to the ASTM D2344 standard.

The impact strength was determined by Charpy’s test using CAEST RESIL 5.5 (impact force of 4 J) for resin and nanocomposites, and Zwick Roell RKP450 (maximum impact force of 300 J) for CFRP. All tests were performed according to the PN-EN ISO 179 standard. Ten unnotched test specimens represented each material. The resin samples were of 10 × 80 mm dimensions and CFRP samples of 15 × 75 mm (width × length) dimensions. The impact strength was calculated from the absorbed energy obtained at the break and according to the PN-EN ISO 179 standard.

The influence of SWCNTs on the glass transition temperature and storage modulus (E′) at room temperature of CFRPs was analysed by Dynamic Mechanical Analysis (DMA). The test was performed using a DMA Q800 (TA Instruments, New Castle, DE, USA) in dual cantilever mode from 0 °C to 180 °C, with the heating rate of 2 °C/min, at the frequency of 1 Hz and with an amplitude of 20 μm. All these tests were performed according to ASTM D7028 with the test samples of 60 mm length and 10 mm width.

## 3. Results and Discussion

### 3.1. Elium^®^/SWCNT Nanocomposites

#### 3.1.1. Viscosity of Elium^®^/SWCNT Mixtures

**Figure 1:**
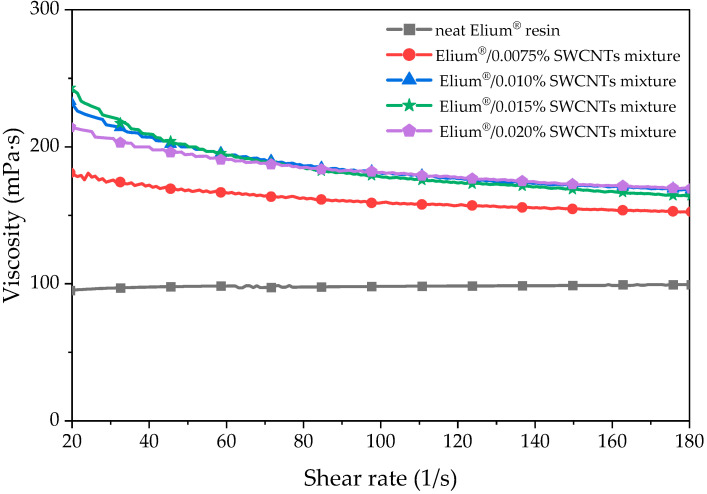
The viscosity of Elium^®^/SWCNT mixtures after the ultrasonication process.

The influence of the SWCNTs’ content on the viscosity of Elium^®^/SWCNT mixtures after the ultrasonication process was examined and the results are presented in Figure 1. Neat Elium^®^ resin possesses the viscosity of 100 mPa·s with Newtonian fluid behaviour, characteristic for fluid polymers. Prepared Elium^®^/SWCNT mixtures showed non-Newtonian fluid behaviour, characterised by a decrease in viscosity with an increasing shear rate. This is a typical shear-thinning behaviour reported in resins with low to moderate loadings of additives [39].

Due to the high surface area of contact between the resin and nanofiller, with a high aspect ratio [18], a significant increase in viscosity was observed for the prepared nanocomposites. The highest change in viscosity was observed between the neat resin and Elium^®^/0.0075 SWCNT nanocomposite. The viscosity increase for nanocomposites with a SWCNT content of more than 0.0075 wt.% is not so noticeable. The increase in viscosity corresponded to the increase in the SWCNT content and reached the maximum value of 169.5 mPa·s for the Elium^®^/0.02% SWCNT mixture. The typical resins used in the infusion process have the viscosity of 100–250 mPa·s, which allows for better permeation of reinforcement [40]. The obtained results indicate that Elium/SWCNT nanocomposites can be used in this manufacturing method.

#### 3.1.2. Electrical Conductivity

**Figure 2:**
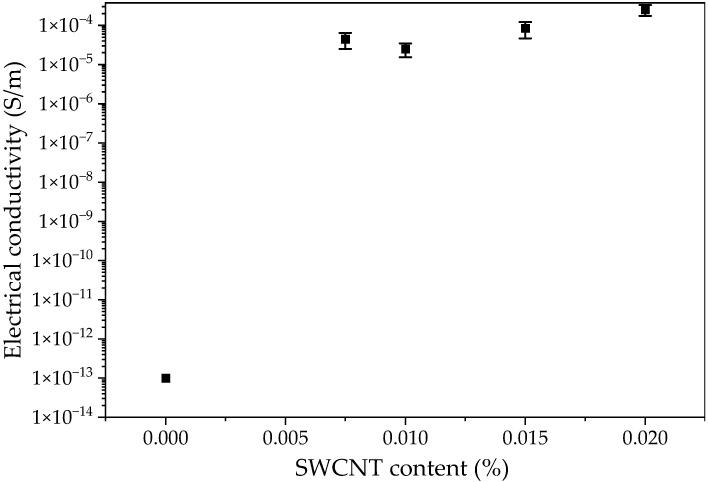
Electrical conductivity of Elium^®^/SWCNT nanocomposites.

The influence of the SWCNTs’ content on the electrical conductivity of manufactured Elium^®^/SWCNT nanocomposites was examined and the results are presented in Figure 2**.** The presented results are mean values.

The range of the device was not sufficient enough to determine the electrical conductivity of the neat Elium^®^ resin and that was the reason why only Elium^®^/SWCNT nanocomposites were examined. The value for neat PMMA of 10^−14^ S/m [41] was used to compare and evaluate the SWCNTs’ influence on electrical conductivity. An increase of about nine orders of magnitude in conductivity was achieved for all nanocomposites. The high aspect ratio of SWCNTs yields significant electrical conductivity improvement with a low level of loading [42] such as in Elium^®^/0.0075 SWCNT nanocomposites. The increase in electrical properties was achieved by the selection of the appropriate dispersion method and parameters. The ultrasonication process time was sufficient to break the nanotubes’ agglomerates, which improved the CNTs’ dispersion in resin [43]. The most significant increase in electrical conductivity was observed for the Elium^®^/0.02 SWCNT nanocomposite from 10^−14^ S/m to 2.52 × 10^−4^ S/m.

Analysing the result obtained for manufactured nanocomposites, it can be estimated that the percolation threshold was below 0.0075 wt.% of the SWCNT for Elium^®^ resin. According to the literature, the percolation threshold was reached for 0.17 wt.% SWCNT/PMMA nanocomposites [44] and 0.084 wt.% of MWCNT/PMMA spin-coated films [45].

### 3.2. Carbon-Fibre-Reinforced Polymers

#### 3.2.1. Microstructure Observations

**Figure 3:**
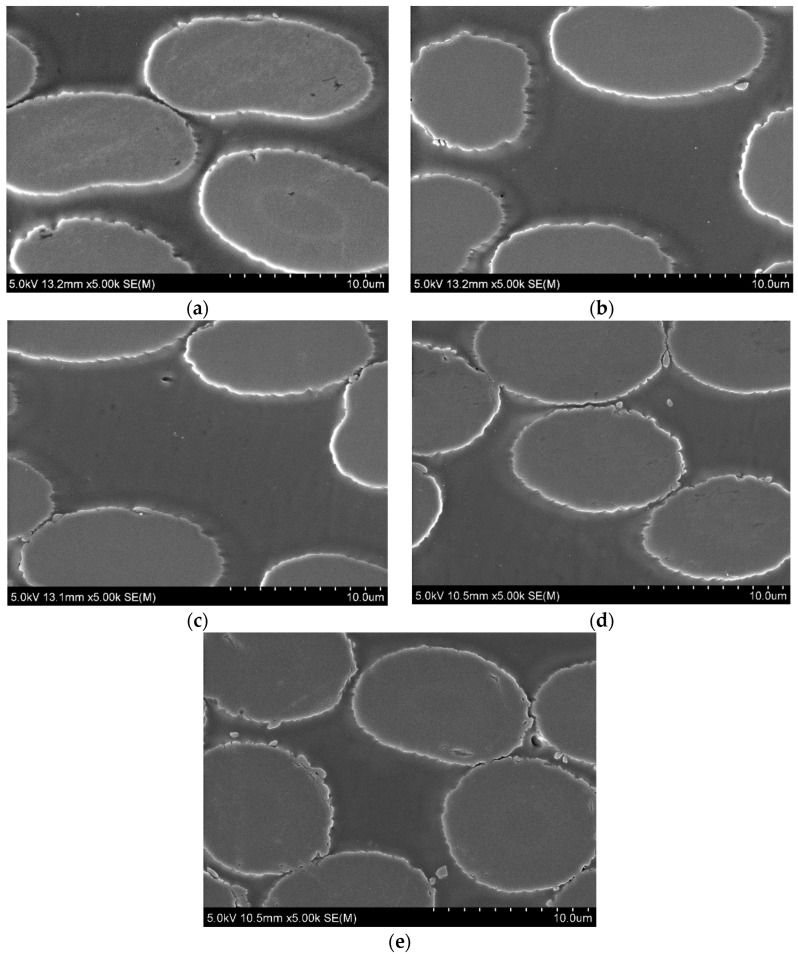
Microstructure observation of CFRP: (**a**) reference; (**b**) Elium^®^/0.0075 SWCNT; (**c**) Elium^®^/0.01 SWCNT; (**d**) Elium^®^/0.015 SWCNT; (**e**) Elium^®^/0.02 SWCNT.

SEM was carried out to investigate the influence of SWCNTs on the adhesion between the carbon fibres and Elium^®^ resin. The microstructure images of the manufactured CFRPs with the visible cross-section of composites are presented in Figure 3. Conducted observations show good adhesion between the polymer matrix and carbon fibres for all manufactured composites. This indicated that the addition of SWCNTs does not affect the interface between the carbon fibre and the matrix. In addition, no voids were observed in the CFRPs’ structure, indicating that the infusion process was carried out correctly.

**Figure 4:**
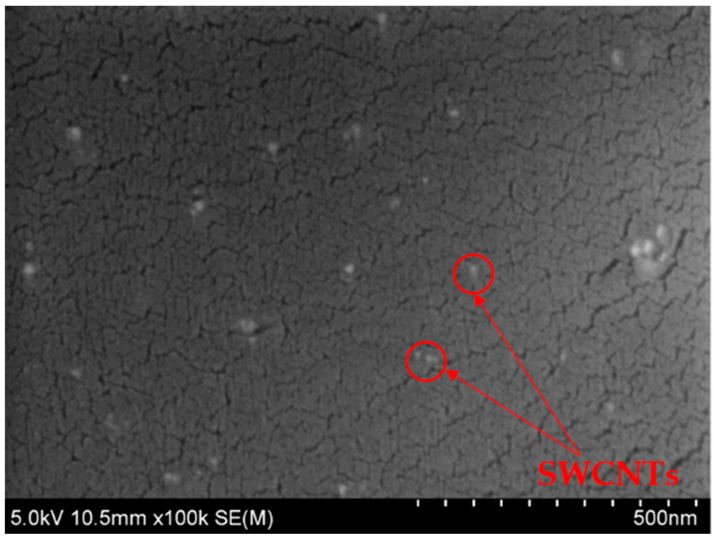
Microstructure of CFRP with 0.02 wt.% of SWCNTs.

Figure 4 presents the microstructure of the CFRP with 0.02 wt.% SWCNT content at a higher magnitude. In this image the uniformly dispersed CNTs in the thermoplastic matrix can be observed. The CNTs are evenly distributed, which indicates that ultrasonication is a suitable method for dispersing the low content of the SWCNTs in Elium^®^ resin. Moreover, such a distribution of CNTs in a polymer matrix results in the formation of conductive paths in the Z direction of composites, and this results in the improved electrical conductivity of the CFRPs [46].

#### 3.2.2. Electrical Conductivity

**Figure 5:**
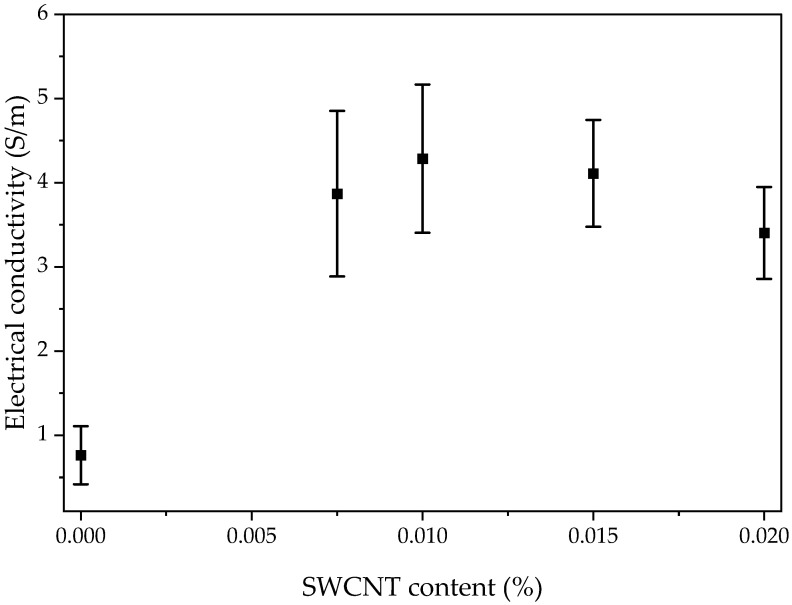
Through-thickness electrical conductivity of CFRPs.

After confirming that the low content of SWCNTs can improve the electrical conductivity of Elium^®^ resin, CFRPs were manufactured by the infusion process. Next, the test was repeated for composite samples obtained from three sections of laminates—the upper, the middle and the bottom part of the laminate. The results are presented in Figure 5. The presented results are mean values.

The results show an approximately 4.5–5.6 times increase in the conductivity of CFRPs with Elium^®^/SWCNT nanocomposites compared to the reference composite. The polymer matrix without modifications acts as an insulator in resin-rich regions between the highly conductive carbon fabric layers [6]. Introducing SWCNTs into Elium leads to the creation of conductive networks in resin-rich regions [47] above the percolation threshold [48]. Due to the high specific ratio and high electrical conductivity of SWCNTs, a low content of filler introduced into the polymer matrix is sufficient to improve the electrical properties of the CFRPs [49]. The modification of resin results in an increase in the electrical conductivity of the CFRPs. The highest increase was observed from 0.76 S/m for the reference laminate to 4.29 S/m for the CFRP with Elium^®^/0.01 SWCNT. The decrease in electrical conductivity was noticed for more than 0.01 wt.% of SWCNTs. It can be related to the higher filtration of CNTs on spaces between fibres during the infusion process; thus, fewer percolation paths can be created [50,51].

#### 3.2.3. Mechanical Properties

The ILSS test was conducted to determine the short-beam strength of the manufactured CFRP and the results are presented in Table 1. The presented results are mean values. In the composites with Elium^®^ resin, additional failure mechanisms such as plastic deformation can occur [8,29,32], which was also observed during the three-point bending test. The results showed that the addition of SWCNTs to the matrix did not affect the short-beam strength of composites in a significant way. The content of the introduced SWCNTs into Elium^®^ was not sufficient to have an impact on the failure mechanisms occurring in the polymer matrix due to the toughening mechanisms of CNTs and the crack bridging effect of the addition of CNTs improving the short-beam strength [52]. However, such a phenomenon occurs with higher CNT content introduced into the polymer matrix. The decrease in short-beam strength was noticeable only for the composite with Elium^®^/0.01 SWCNT. This indicates that the addition of nanofiller did not affect the adhesion between the matrix and carbon fibres, which was also confirmed during microstructure observations [53,54].

**Figure 6:**
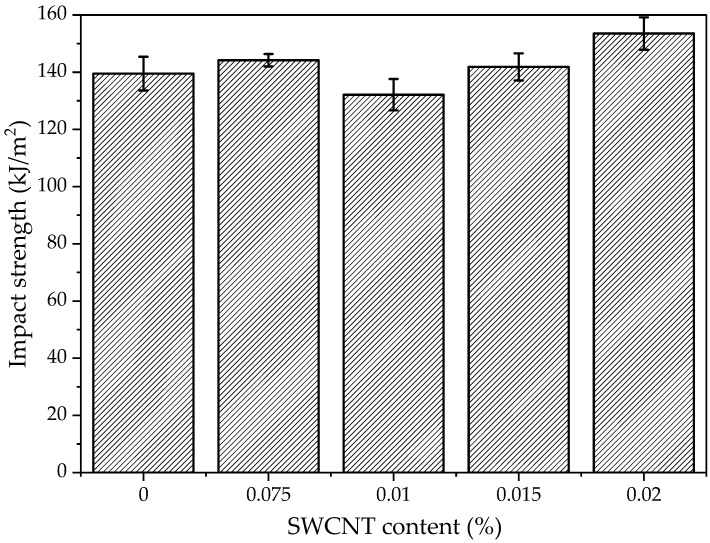
Impact strength of Elium^®^ resin and Elium^®^/SWCNT nanocomposites.

The Charpy’s impact test was conducted to determine the influence of SWCNTs on impact strength and the results are presented in Figure 6. The presented results are mean values. These results show that below 0.02 wt.% of SWCNTs introduced into Elium^®^ resin, the influence of the nanofiller on impact strength is minimal. The increase in impact strength after the addition of CNTs is related to the factors such as CNTs’ pullout and bridging, which contribute to the fracture toughness of composites [55]. Moreover, during longitudinal loading, the matrix of composites plays a dominant role in energy absorption and energy redistribution to fibres [56]. The results show that at least 0.02 wt.% of SWCNTs must be added to the polymer matrix to obtain a reinforcing effect. A 10% increase in impact strength was obtained for the highest fraction of SWCNTs.

#### 3.2.4. Dynamic Mechanical Analysis

**Figure 7:**
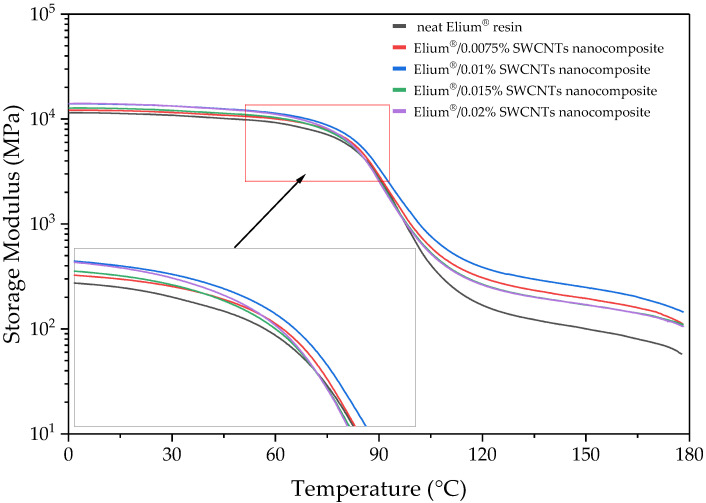
Dynamic Mechanical Analysis results of CFRP, storage modulus (G′).

**Figure 8:**
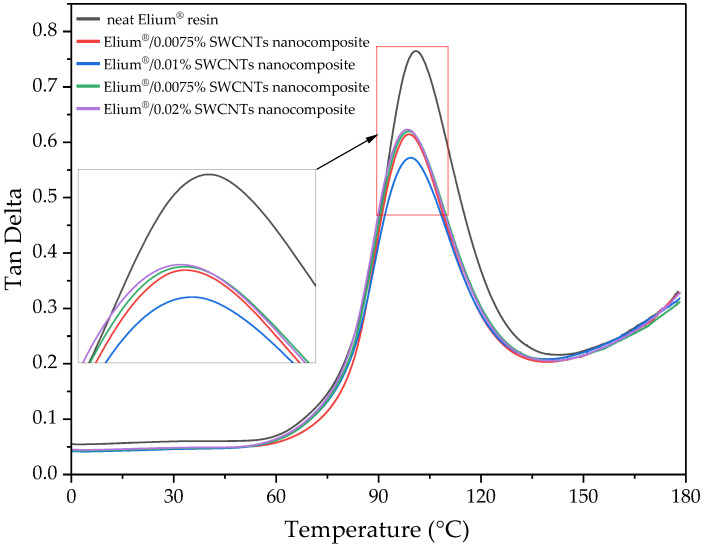
Dynamic Mechanical Analysis results of CFRP, tan delta (tan δ).

The thermomechanical properties of the manufactured composites were investigated by conducting DMA. Based on the obtained results, the storage modulus (E′) and the dampening parameter (tan δ), defined as the ratio of the loss to storage modulus, were analysed. The plots of these parameters depending on temperature are presented in Figure 7 and Figure 8, respectively. The storage modulus (E′) defines the elastic behaviour of composites, while the dampening parameter (tan δ) evaluates energy dissipation in the material. The addition of the nanofiller increased the storage modulus (Table 2) because of the molecular interactions between the polymer matrix and the SWCNTs [57]. The highest storage modulus value at room temperature was obtained for the CFRPs with 0.02 wt.% of SWCNTs. As shown in Figure 7, the addition of SWCNTs does not significantly affect the initial shape of the curve for storage modulus to the temperature of about 95 °C. Above this temperature, the storage modulus of the CFRPs with neat resin decreases faster than for the composites with the addition of SWCNTs. This is due to the high stiffness of SWCNTs [14,15] and the interaction between the matrix and the nanofiller. Although the changes in the glass transition temperature for all tested CFRPs are insignificant (Table 2), even such a small SWCNT content can slow down the decrease in elastic properties.

The addition of SWCNTs causes a decrease in the peak intensity of tan δ. In the composites with Elium^®^ resin, additional failure mechanisms such as plastic deformation can occur [29,32]. However, SWCNTs with high mechanical properties and high stiffness can hinder the occurrence of such mechanisms. Moreover, the nanofiller causes a shift to the left side of the tan delta curves (Figure 8). The shift of the tan δ peak to a lower temperature can be associated with a lower degree of polymerisation of Elium^®^ resin. The SWCNTs can act as inhibitors of the free radical polymerisation process.

## 4. Conclusions

In this study, a novel solution to address the problems of CFRPs with thermoset matrices such as brittleness, a difficult recycling process and low electrical conductivity has been proposed. CFRPs with the novel thermoplastic resin Elium^®^, modified with SWCNTs, were manufactured by the infusion method. The mechanical and thermomechanical properties and electrical conductivity of composites with low SWCNT content (from 0.0075 wt.% to 0.02 wt.%) were investigated. Microstructure observations were conducted to investigate the influence of the nanofiller on the interface of carbon fibre/matrix and the dispersion of the SWCNTs.

Introducing SWCNTs into thermoplastic resin results in a viscosity increase due to molecular interactions between the polymer matrix and the nanofiller. Comparing the results, the addition of 0.02 wt.% of SWCNTs increased the viscosity to a value of 169.5 mPa·s, whereas that of the reference sample was 100 mPa·s. Furthermore, modified nanocomposites with such characteristics can be used in the resin infusion process. Moreover, introduced nanotubes create percolation paths. This resulted in an increase of about nine orders of magnitude in electrical conductivity through the thickness of laminates.

Next, the CFRPs were manufactured by the resin infusion process. The microscope observations show evenly dispersed nanotubes in the polymer matrix. Moreover, no defects, which can decrease mechanical properties, were observed. The evenly distributed SWCNTs introduced into the matrix created conductive paths in resin-rich regions between carbon fabric layers. This resulted in a 4.5–5.6 times increase in the conductivity of CFRPs with Elium^®^/SWCNT nanocomposites compared to the reference composite.

Despite the significant increase in electrical conductivity, low concentrations of SWCNTs were insufficient to have a significant impact on the short-beam strength and impact resistance of CFRPs. Mechanisms such as CNTs pullout and crack bridging were dominated by plastic deformation of the polymer matrix during the tests. The obtained results were comparable for each tested CFRP and only for the composite with the addition of 0.02 wt.% of SWCNTs was a 10% increase in impact resistance compared to the reference observed. The DMA test indicated that a low SWCNT content introduced into the matrix has an insignificant effect on the glass transition temperature of CFRPs. However, it is noteworthy that the introduced carbon nanotubes increased the storage modulus of the CFRPs at room temperature. The highest increase of 20% was observed for the composite with the addition of 0.02 wt.% of SWCNTs. Moreover, CFRPs with a modified matrix exhibit a slower loss of the elastic properties of composites due to the high stiffness of SWCNTs. Additionally, the shift in the damping parameter indicates that SWCNTs act as inhibitors of the free radical polymerisation process.

Taking into account the obtained results, it has been proved that introducing a low content of SWCNTs into thermoplastic resin Elium^®^ improves the electrical conductivity of CFRPs. However, the amount of the introduced nanofiller was insufficient to activate the toughening mechanism and CNTs’ crack bridging to enhance the mechanical performance of composites.

## Figures and Tables

**Table 1:** Flexural properties of manufactured composites.

SWCNT Content (%)	F^sbs^ (MPa)
0	29.3 ± 0.8
0.0075	28.7 ± 2.1
0.0100	26.3 ± 2.8
0.0150	29.5 ± 1.9
0.0200	30.2 ± 1.7

**Table 2:** Storage modulus (E′) at room temperature and glass transition temperature (T_g_) of CFRP determined from the tan delta.

SWCNT Content (%)	Glass Transition Temperature (°C)	Storage Modulus @ 25 °C (MPa)
0	101	11,300
0.0075	99	11,750
0.0100	99	13,544
0.0150	99	12,293
0.0200	99	13,572

## Data Availability

All data are available in the main text.
